# *CNTN6* mutations are risk factors for abnormal auditory sensory perception in autism spectrum disorders

**DOI:** 10.1038/mp.2016.61

**Published:** 2016-05-10

**Authors:** O Mercati, G Huguet, A Danckaert, G André-Leroux, A Maruani, M Bellinzoni, T Rolland, L Gouder, A Mathieu, J Buratti, F Amsellem, M Benabou, J Van-Gils, A Beggiato, M Konyukh, J-P Bourgeois, M J Gazzellone, R K C Yuen, S Walker, M Delépine, A Boland, B Régnault, M Francois, T Van Den Abbeele, A L Mosca-Boidron, L Faivre, Y Shimoda, K Watanabe, D Bonneau, M Rastam, M Leboyer, S W Scherer, C Gillberg, R Delorme, I Cloëz-Tayarani, T Bourgeron

**Affiliations:** 1 Human Genetics and Cognitive Functions Unit, Institut Pasteur, Paris, France; 2 CNRS UMR 3571: Genes, Synapses and Cognition, Institut Pasteur, Paris, France; 3 Université Paris Diderot, Sorbonne Paris Cité, Human Genetics and Cognitive Functions, Paris, France; 4 Imagopole, Citech, Institut Pasteur, Paris, France; 5 Institut Pasteur, Unité de Microbiologie Structurale, Paris, France; 6 CNRS UMR 3528, Paris, France; 7 INRA, Unité MaIAGE, UR1404, Jouy-en-Josas, France; 8 Assistance Publique-Hôpitaux de Paris, Child and Adolescent Psychiatry Department, Robert Debré Hospital, Paris, France; 9 Centre for Applied Genomics, Program in Genetics and Genome Biology, Hospital for Sick Children, Toronto, ON, Canada; 10 Centre National de Génotypage, Evry, France; 11 Eukaryote Genotyping Platform, Genopole, Institut Pasteur, Paris, France; 12 Assistance Publique-Hôpitaux de Paris, ENT and Head and Neck Surgery Department, Robert Debré Hospital, Paris-VII University, Paris, France; 13 Département de Génétique, CHU Dijon et Université de Bourgogne, Dijon, France; 14 Department of Bioengineering, Nagaoka University of Technology, Nagaoka, Japan; 15 Département de Biochimie et Génétique, Centre Hospitalier Universitaire, Angers, France; 16 Department of Clinical Sciences in Lund, Lund University, Lund, Sweden; 17 Gillberg Neuropsychiatry Centre, University of Gothenburg, Gothenburg, Sweden; 18 INSERM U955, Psychiatrie Translationnelle, Créteil, France; 19 Université Paris Est, Faculté de Médecine, Créteil, France; 20 Assistance Publique-Hôpitaux de Paris, DHU Pe-PSY, H. Mondor Hospital, Department of Psychiatry, Créteil, France; 21 FondaMental Foundation, Créteil, France; 22 McLaughlin Centre, Department of Molecular Genetics, University of Toronto, Toronto, ON, Canada

## Abstract

Contactin genes *CNTN5* and *CNTN6* code for neuronal cell adhesion molecules that promote neurite outgrowth in sensory-motor neuronal pathways. Mutations of *CNTN5* and *CNTN6* have previously been reported in individuals with autism spectrum disorders (ASDs), but very little is known on their prevalence and clinical impact. In this study, we identified *CNTN5* and *CNTN6* deleterious variants in individuals with ASD. Among the carriers, a girl with ASD and attention-deficit/hyperactivity disorder was carrying five copies of *CNTN5*. For *CNTN6*, both deletions (6/1534 ASD vs 1/8936 controls; *P*=0.00006) and private coding sequence variants (18/501 ASD vs 535/33480 controls; *P*=0.0005) were enriched in individuals with ASD. Among the rare *CNTN6* variants, two deletions were transmitted by fathers diagnosed with ASD, one stop mutation CNTN6^W923X^ was transmitted by a mother to her two sons with ASD and one variant CNTN6^P770L^ was found *de novo* in a boy with ASD. Clinical investigations of the patients carrying CNTN5 or CNTN6 variants showed that they were hypersensitive to sounds (a condition called hyperacusis) and displayed changes in wave latency within the auditory pathway. These results reinforce the hypothesis of abnormal neuronal connectivity in the pathophysiology of ASD and shed new light on the genes that increase risk for abnormal sensory perception in ASD.

## Introduction

Autism spectrum disorders (ASD) are a heterogeneous group of disorders with different causes, phenotypic outcomes and ages of onset.^1, 2, 3^ The diagnosis of ASD is based on impairments in reciprocal social communication and restricted, repetitive patterns of behaviors. In addition to these behavioral phenotypes, sensory-motor peculiarities are often present and are now included as one of the possible qualifying behavioral symptoms for a diagnosis of ASD.^4^ These include an apparent indifference to pain/heat/cold, adverse response to specific sounds (for example, hyperacusis) or textures, excessive sensation when smelling or touching objects, and fascination with lights or spinning objects.^3, 5^ Individuals with ASD also exhibit alterations in sensory processing, including difficulties in the integration of information across different sensory modalities.^6, 7^ In addition, motor control abnormalities—for example, poor manual dexterity and coordination—are frequently reported in patients.^8^ It has been proposed that these sensory-motor problems—especially those affecting the auditory pathway—might lead to communication impairments and subsequently to autism.^9, 10, 11, 12, 13, 14, 15, 16, 17^ Interestingly, mutations of genes related to hearing loss were found in subjects with ASD,^18^ but to date, no gene has been directly associated with sensory-motor impairments in ASD and the causes of such clinical features remain unknown.

Genetic studies have demonstrated that hundreds of genes may be involved in the pathogenesis of ASD.^19, 20, 21^ The genetic variations include copy-number variants (CNVs) and single-nucleotide variants (SNVs), which can be inherited or *de novo*.^19^ In a subset of patients, ASD appear to be a monogenic trait involving a single mutation with high penetrance.^22, 23^ However, in a majority of patients, the heritability of ASD is considered polygenic with a combination of inherited rare and common variants.^19, 24^ In these cases, risk variants may not fully segregate with the trait and are usually present in a small subset of patients.^21^ At least three main biological pathways have been associated with ASD: chromatin remodeling, mRNA translation and synaptic function.^25^

Among the candidate genes for ASD, Contactin *CNTN5* and *CNTN6* genes code for neural cell adhesion proteins that promote neurite outgrowth and synaptogenesis.^26, 27, 28, 29, 30, 31^ CNTNs are attached to the cell membrane by a glycosylphosphatidyl inositol anchor and can be found in two active forms, membrane-bound and secreted.^26, 27^ They contain six immunoglobulin-like (Ig) domains followed by four fibronectin type III (FNIII) domains. In mice, CNTN5 (also named NB-2) and CNTN6 (also named NB-3) are key proteins for the development of sensory-motor pathways.^32, 33, 34, 35^ CNTN5 contributes to the development of glutamatergic neurons in the auditory brainstem, from the ear through the inferior colliculus to the cortex.^34^ Mice lacking CNTN5 present with increased auditory brainstem response (ABR) wave latencies.^34^ CNTN5 is also expressed in mouse retinal neurons^36^ and at high levels in the human lingual gyrus, a brain region involved in visual processing.^37^ CNTN6 is regulated by T-Brain-1,^38^ an ASD-risk protein, and interacts with cell adhesion molecule L1-like,^33^ another protein associated with intellectual disability (ID) and language difficulties.^39^ CNTN6 also interacts with NOTCH1 to produce oligodendrocytes from progenitor cells^40, 41, 42^ and is highly expressed in the inferior colliculus and in the cerebellum.^32^ CNTN6 is crucial for appropriate orientation of dendrite growth in mouse cortical pyramidal neurons,^33^ and for synapse formation in the cerebellum.^43^ Auditory function has not yet been investigated in mice lacking CNTN6, but they display impaired motor coordination.^32^

Several lines of evidence suggest that mutations of CNTNs and their binding partners, the Contactin-associated proteins (CNTNAPs), are risk factors for ASD.^26, 27, 44^ First, heterozygous deletions of *CNTN4*, *CNTN5* or *CNTN6* (refs 45, 46, 47, 48, 49, 50, 51, 52) have been identified in patients with neuropsychiatric disorders such as ASD and ID. In addition, individuals with *CNTNAP2* mutations display ID and epilepsy when mutations are homozygous^53^ or higher risk for ASD and/or language impairments when mutations are heterozygous.^54, 55, 56, 57, 58^ Finally, heterozygous deletions of *CNTNAP4* and *CNTNAP5* have been identified in a few cases of ASD.^56, 58, 59, 60, 61, 62^ Recently, a large mutation screen has detected *de novo* mutations of *CNTN6* and *CNTNAP4* in two unrelated patients with ASD, but no significant association between *CNTN* and *CNTNAP* rare SNVs and ASD.^63^ Nevertheless, the authors of this study did not exclude that deleterious CNTN/CNTNAP variants could increase the risk of ASD in a subset of patients and were soliciting for functional studies to better ascertain the impact of the variants.^63^

In our study, we assessed the frequency of CNVs and SNVs affecting *CNTN5* and *CNTN6* in patients with ASD. We then evaluated the functional effects of the SNVs on neurite outgrowth using cultured neurons and addressed the molecular issues of those mutations on protein structure. Finally, given the involvement of *CNTN5* and *CNTN6* in the development of sensory-motor neuronal pathways, a clinical exploration of motor coordination and ABR was conducted in patients carrying *CNTN5* or *CNTN6* variants, and their relatives.

## Materials and methods

### Patients and controls

The ASD diagnosis was based on clinical expert assessment including the Autism Diagnostic Interview–Revised (ADI-R)^64^ and the Autism Diagnostic Observation Schedule.^65^ In a few cases, the Diagnostic Interview for Social and Communication Disorders (DISCO-10)^66^ was used instead of the ADI-R. Intellectual quotient was measured using an age-appropriate Wechsler scale. For the most severe and/or non-verbal patients, the Raven's Standard Progressive Matrices and the Peabody Picture Vocabulary test were used. The cohorts recruited by the PARIS (Paris Autism Research International Sibpair) study are described in Supplementary Table 1. Families AUDIJ001 and AUDIJ002 carrying *CNTN5* CNVs were not part of our initial cohort of patients and therefore were not included in the association analysis (Supplementary Figure 1). Gross and fine motor coordination abilities were assessed during the neurological exam and with the Developmental Coordination Disorder Questionnaire (DCD-Q).^67^ The local Institutional Review Boards at Hôpital Pitié-Salpêtrière (Paris, France) and University of Gothenburg (Sweden) approved the study. Written informed consent was obtained from all participants. For the patients who were unable to consent for themselves, a parent or legal guardian consented to the study on their behalf.

### Auditory brainstem response audiometry

An experienced Ear-Nose-Throat specialist examined some of the patients carrying CNTN5 or CNTN6 variants and their first-degree relatives. The exploration included otoscopic examination, tympanogram and a measurement of the stapedian ipsilateral reflexes. Recording of the ABR was performed using the Biologic Navigator-Pro Evoked Potential System (Natus Medical, Mundelein, IL, USA). We used the Wilcoxon non-parametric test to detect statistical difference in wave latency between carriers and non-carriers of CNTN5 or CNTN6 variants, and between affected and non-affected subjects.

### Genetic analyses

For CNV detection, 1534 unrelated individuals with ASD (901 from Pinto *et al.*^68^ and 633 from our cohort) and 8936 controls were analyzed using Illumina SNP arrays (Supplementary Tables 1 and 2). Two CNV detection algorithms, PennCNV and QuantiSNP, were used and all samples met stringent quality control criteria as described.^68^ For SNV detection, 429 individuals (212 independent patients with ASD and 217 controls) were screened for mutations in all exons of *CNTN5* and *CNTN6* using Sanger sequencing (Supplementary Tables 3 and 4). For replication, we had access to the results of whole-genome sequence from a sample of 289 individuals with ASD (200 trios and 89 sib pairs).^18^ The whole-genome sequence was obtained as previously described.^18^ The rare variants were defined as in Murdoch *et al.*:^63^ seen in either cases or controls exclusively, missense, nonsense, splice site, or start or stop codon disruptions with a frequency of less than 1% in all populations from the general European (Non-Finnish) population from ExAC (http://exac.broadinstitute.org/). To estimate the frequency of individuals sequenced for CNTN5 or CNTN6 variants in ExAC, we used the median of the number of alleles sequenced divided by a factor of 2 (CNTN5=32858; CNTN6=33263) and we assumed that a single individual was carrying only one rare variant. Variants were considered deleterious when they were predicted as damaging by at least two of these five criteria: CADD Phred score≥20, SIFT≤0.05, PolyPhen2≥0.453, Mutation Assessor≥2, vertebrate PhyloP≥2. We used a one-sided Fisher's exact test or a *χ*^2^ test to test for enrichment in CNVs or SNVs in patients compared with controls and the G*power software (http://www.gpower.hhu.de/) to estimate the achieved power of our analyses. For the multiple hits in known ASD-risk genes, we identified exonic CNVs and deleterious SNVs in Class I-III genes from Yuen *et al.*,^18^ TADA genes from Sanders *et al.* ^69^ or genes from the SFARI database (9 November 2015; https://gene.sfari.org/autdb/Welcome.do). The overlap between the database is illustrated in Supplementary Figure 7 and the complete list of genes is indicated in Supplementary Table 10. For SNVs, we only considered those with a minor allele frequency of less than 1% in the general population from ExAC and from the 1000 genomes.

### Cell culture procedures and *in vitro* analysis of neurite outgrowth

Experiments were performed according to the standardized co-culture assay and automated quantification method, which we published previously.^31^ Primary rat cortical neurons were prepared from newborn (P0-P1) Sprague–Dawley rats, plated at a density of 4x10^5^ cells per ml and cultured for 6 days before adding the HEK293 cells. HEK293 cells were cultured in Minimum Essential Medium containing 100 U ml^−1^ penicillin, 100 μg ml^−1^ streptomycin, 2 mm glutamine (Invitrogen, Life Technologies SAS, Saint-Aubin, France) and 10% fetal calf serum (ref. CVFSVF00-01, Eurobio, Courtaboeuf, France). HEK293 cells were transfected with rat CNTN6 cDNAs cloned in pcDNA3.1 vector (CNTN6 GenBank accession number: D87248) using the jet PRIME® kit (POL114-15 Polyplus-transfection SA, Illkirch, France). After transfection, 2–3x10^5^ cells were collected and seeded on top of the neurons in culture. The percentage of transfected cells was very similar in all experiments and corresponded to 50% of the HEK cells. HEK293 and neurons were co-cultured for 2 days before fixation with 4% paraformaldehyde. The secreted CNTN6 were at an estimated concentration of 100 ng ml^−1^.^31^ Western blots and immunofluorescence labeling on HEK293 cells were performed 2 days after transfection as previously described.^31^ Mouse anti-rat NB-3 (2F7) monoclonal antibodies were used at a dilution of 1/500.^34, 43, 70^ Cells in co-cultures were incubated with the primary mouse anti-MAP2 antibody (ref. MAB3418, Millipore, Molsheim, France) at a dilution of 1/500. The secondary antibody was an Alexa Fluor 594 Goat Anti-Mouse IgG (H+L) used at a dilution of 1/200 (A11005, Molecular Probes, Life Technologies). After washing, coverslips were mounted on glass slides with ProLong antifade reagent with DAPI (Invitrogen, Life Technologies). Fluorescence mosaic images were acquired with an inverted microscope Axio Observer.Z1 (Carl Zeiss, Le Pecq, France). Constructs carrying a non-synonymous variant were generated by site-directed mutagenesis of the wild-type rat *CNTN6* cDNA sequence using the QuikChange XL II Site-Directed Mutagenesis Kit from Agilent (Santa Clara, CA, USA). Primers were designed using Agilent's QuikChange Primer Design program (Supplementary Table 5). Mutated plasmids were then purified using NucleoBond Xtra Maxi EF from Macherey-Nagel, and sequenced.

### Immunoglobulin and fibronectin domains: homology modeling

CNTN6^Ig1-4^ were homology modeled, using the model-building software Modeller (mod9v7) from the solved X-ray template of mouse CNTN4^Ig1-4^ (Protein Data Bank ID: 3KLD). Each CNTN6 variant was introduced in the.*pir* alignment file of the corresponding wild-type protein. Models (*N*=50) were generated using Modeller, to satisfy the spatial restraints issued from the alignment with the target protein mouse CNTN4^Ig1-4^.^71^ Models with the lowest score function values and best stereochemistry, checked by Molprobity (http://molprobity.biochem.duke.edu/), were then subjected to energy minimization using CharmM forcefield with the backbone constrained (DS2.5; Accelrys, San Diego, CA, USA).^72^ Similarly, each FNIII sequence was three-dimensionally aligned using Espript (http://espript.ibcp.fr/ESPript/) with its template selected using HHPred server (http://toolkit.tuebingen.mpg.de/hhpred). For each FNIII building model, 100 homology models were generated using Modeller. After minimization with CharmM, all resulting WT and variant models were manually analyzed using Pymol (https://www.pymol.org/).

## Results

### Frequency of CNTN5 and CNTN6 variants in ASD and controls

**Figure 1:**
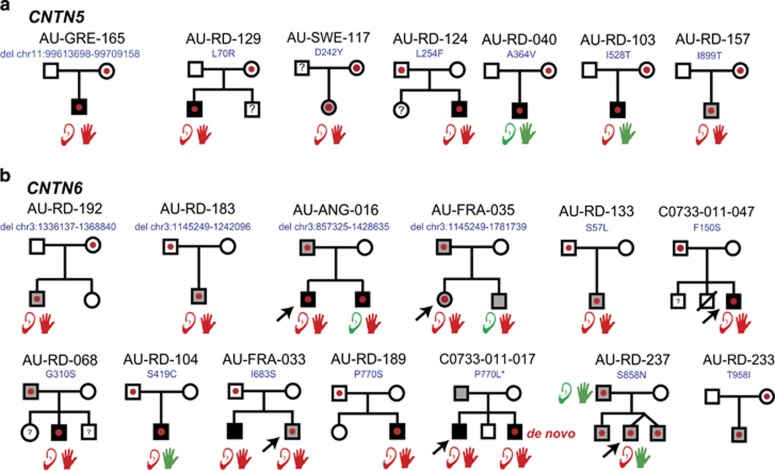
Families carrying *CNTN5* (**a**) and *CNTN6* (**b**) exonic CNVs or rare SNVs. *CNTN5* and *CNTN6* CNVs and SNVs are represented by dots. Black-filled symbols indicate a diagnosis of ASD with intellectual disability, whereas gray-filled symbols indicate a diagnosis of ASD without intellectual disability. In multiplex families, an arrow indicates the proband. Based on the ADI-R and clinical examination, normal (green) or abnormal (red) hypersensitivity to sounds and motor coordination ability are indicated by a schematic ear and hand, respectively. The absence of ear/hand means that information was not available. The locations of all CNVs identified in this study are indicated (hg19) and shown in Supplementary Figures 2 and 3. All families with *CNTN5*- or *CNTN6*-coding SNVs are shown in Supplementary Figure 4. ASD, autism spectrum disorder; CNV, copy-number variants; SNV, single-nucleotide variants.

**Figure 2:**
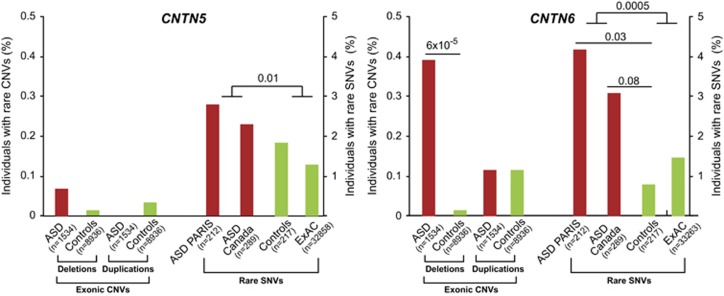
Frequency of *CNTN5* and *CNTN6* exonic CNVs and rare SNVs identified in patients with ASD and in controls. Differences in frequency of carriers of *CNTN5* or *CNTN6* CNVs or SNVs between patients and controls were calculated using a Fischer's exact test. The rare SNVs were defined as in Murdoch *et al.:*^63^ seen in either cases or controls exclusively, missense, nonsense, splice site, or start or stop codon disruptions with a frequency of less than 1% in the general European (Non-Finnish) population from ExAC (http://exac.broadinstitute.org/). ASD, autism spectrum disorder; CNV, copy-number variants; SNV, single-nucleotide variants.

We first screened for CNVs affecting exons of *CNTN5* and *CNTN6* in our cohort of 633 individuals with ASD. We identified one patient with a deletion of *CNTN5* and four patients with a deletion of *CNTN6* (Figures 1 and 2; Supplementary Figures 2 and 3). None of the patients had a second deleterious *CNTN5/6* variant on the remaining allele. In the cohort of the Autism Genome Project from Pinto *et al.*,^68^ we observed 2 *CNTN6* deletions and 2 *CNTN6* duplications out of 901 patients with ASD. In our sample of 8936 individuals from the general population (Supplementary Table 2), we observed 1 deletion and 3 duplications of *CNTN5* as well as 1 deletion and 12 duplications of *CNTN6*. Overall, *CNTN6* deletions were more frequent in patients compared with controls (ASD 6/1534 (0.39%) vs controls 1/8936 (0.01%); *P*=6 × 10^−5^).

We also had access to the phenotypes of the patients from the Brain & Body Genetic Resource Exchange (BBGRE version 3.0; https://bbgre.brc.iop.kcl.ac.uk/) database that includes 5891 patients (776 with ASD). We found a total of 14 *CNTN6* deletions out of the 5891 patients (Supplementary Table 11) and a significant excess of *CNTN6* deletions in patients with ASD (6/776; 0.77% *P*=0.02). This excess of *CNTN6* deletions in ASD is even more significant when only small deletions (<500 kb) are considered. There are 7 small *CNTN6* deletions out of 5891 patients listed in BBGRE version 3.0 and 6 out of 776 in patients with ASD (*P*=0.002). Finally, in the Decipher database (https://decipher.sanger.ac.uk/index), a total of 47 *CNTN6* deletions are listed among 18 506 patients (0.25%). In contrast to the BBGRE database, the phenotype for autism is rarely indicated, but several patients carrying *CNTN6* deletions have cognitive impairments, ID or ASD (Supplementary Table 12).

In our cohort of patients, we had access to the DNA from the parents and all CNVs were inherited. Interestingly, two father carriers of a *CNTN6* deletion were diagnosed with ASD. After this initial screen, our collaborators (ALMB, LF) identified two additional families with *CNTN5* CNVs (Supplementary Figure 1). In family AUDIJ001, the mother, who had ASD, transmitted a deletion of *CNTN5* to her daughter with specific language impairment. In family AUDIJ002, a girl with ASD and attention-deficit/hyperactivity disorder carried five copies of *CNTN5* transmitted by her mother, who had specific learning disorder (reading).

**Figure 3:**
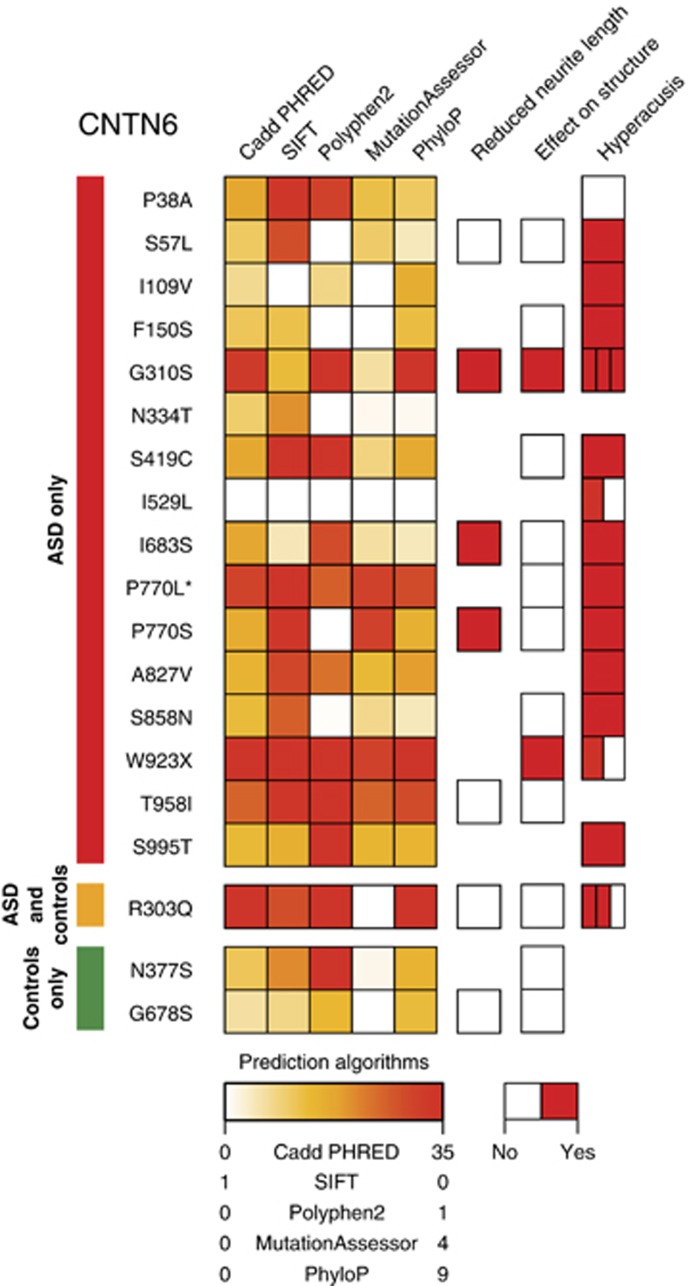
Functional impact of the CNTN6 variants. The predicted scores from five prediction algorithms are shown as a gradient for each mutation. The effect of each variant on neurite length, on the structure of the CNTN6 protein and the clinical observation of hypersensitivity to sounds (hyperacusis) are also indicated. The Divided squares represent multiple autism spectrum disorder (ASD) individuals carrying the same mutation. Missing squares represent unavailable information. The star indicates a *de novo* mutation.

We then sequenced all coding exons of the *CNTN5* and *CNTN6* genes in 429 individuals, including 212 independent patients with ASD and 217 controls from France and Sweden (Figures 1 and 2, Supplementary Figure 4 and Supplementary Tables 6 and 7). For *CNTN5*, we observed private variants in 5/212 (2.35%) patients compared with 4/217 (1.84%) controls (*P*=0.36). For *CNTN6*, we observed 9/212 (4.24%) individuals with ASD carrying a private variant compared with 2/217 (0.92%) controls (*P*=0.03, odds ratio=4.68, 95% confidence interval=1–21.8). Among the affected carriers, we observed a *de novo* CNTN6^P770L^ variant predicted as deleterious by all algorithms (Figure 3). We then confirmed the frequency of rare variants of *CNTN5* and *CNTN6* in an independent cohort of 289 patients with ASD from Canada (Supplementary Figure 5). We found very similar frequencies for *CNTN5* (7/289; 2.4%) and *CNTN6* (9/289; 3.1%) rare variants in the Canadian cohort of patients with ASD compared with our patients with ASD. In one family, two affected brothers with ASD carried a CNTN6^W923X^ stop mutation transmitted by the mother.

In summary, both *CNTN6* CNVs and rare SNVs were more frequent in patients with ASD compared with the general population. Given our sample and effect sizes, our achieved statistical power was 92% for the CNVs, but only 62% for the SNVs. To increase our statistical power for the SNVs, we used the sequencing data obtained in >30 000 individuals from the ExAC database (Supplementary Figure 6). The frequency of rare *CNTN6* variants in individuals from the general European population from ExAC (533/33263; 1.6%) was not significantly different from our controls (2/217; 0.92% *P*=0.6). Using the cohorts from PARIS and Canada and the ExAC data set, the enrichment of rare *CNTN6* variants in individuals with ASD compared with the general population was highly significant (ASD: 18/501; 3.59% controls: 535/33 480; 1.6% *P*=0.0005) and with an achieved power to observe such a difference of 87%.

### Additional ASD-risk gene deleterious variants in patients carrying CNTN6 variants

As often found in genetic studies of ASD, the risk variants do not always co-segregate with the phenotypes. We therefore investigated whether individuals with ASD carrying a rare *CNTN6* variant had other rare (minor allele frequency<1% in 1000 genomes or ExAC) and deleterious (considered as damaging by two algorithms) variants in genes known to be associated with ASD (for the ASD-risk genes see Supplementary Figure 7 and Supplementary Table 10). For all patients carrying CNTN6 variants, we searched for rare CNVs and screened for exonic SNVs in *CNTN3*, *CNTN4* and *CNTNAP2*. Finally, we analyzed the data from whole-exome sequencing (*N*=9 families) and whole-genome sequencing (*N*=11 families) to identify deleterious variants in known ASD-risk genes. Among the CNVs that we identified (Table 1), we found a maternal deletion of 29 kb including the fifth exon of the X-linked Duchenne muscular dystrophy (*DMD)* gene in a patient carrying a paternal *CNTN6* duplication, a paternal duplication of 285 kb including all exons of Nephrocystine 1 (*NPHP1*) gene in a patient carrying a maternal *CNTN6* deletion, and a paternal duplication of 873 kb including the first exon of the glutamate receptor ionotropic delta 2 gene (*GRID2*) in a patient carrying a *de novo* CNTN6^P770S^ variant. For the families with whole-exome/genome sequencing data, we found a X-linked stop mutation of the Ras-Associated *RAB39B* gene causing X-linked ID^73^ in two brothers carrying the stop mutation CNTN6^W923X^. A paternally transmitted frameshift mutation of the histone deacetylase *HDAC4* gene associated with ID^68^ was identified in a patient carrying the CNTN6^I529L^ variant. In summary, although our study is underpowered to identify specific biological pathways mutated in patients carrying CNTN6 variants, we could detect several additional rare deleterious variants in known risk genes for ID or ASD.

### Functional impact of CNTN6 variants

*CNTN6* is known to enhance neurite outgrowth both *in vitro*^31^ and *in vivo*.^35^ In order to estimate the functional impact of CNTN6 variants, we used five prediction algorithms, an *in vitro* assay for the effect of CNTN6 variants on neurite outgrowth and a homology modeling of the protein (Figure 3 and Supplementary Figures 8 and 9). Several CNTN6 variants identified in this study were considered deleterious by at least two algorithms (Figure 3). Using an *in vitro* assay, we showed that some variants (CNTN6^G310S^, CNTN6^I683S^, CNTN6^P770S^) could affect the promoting effect of CNTN6 on neuritogenesis, whereas others did not (CNTN6^S57L^, CNTN6^T958I^, CNTN6^R303Q^, CNTN6^G678S^; Figure 3 and Supplementary Figure 8). Based on the homology model of the CNTN6 protein structure, the variant CNTN6^G310S^, observed in the French and Canadian cohorts of patients, might induce a molecule distortion (Supplementary Figures 8 and 9). The CNTN6^T958I^ variant located in the fibronectin domains and identified in a patient with ASD, corresponds to the position of a critical amino acid (L1046) of the neogenin, a receptor of the axon guidance molecule netrin and a binding partner of the repulsive guidance molecule family members.^74^ For this CNTN6^T958I^ variant, we observed a putative secreted dimeric form suggested by the presence of an additional band of twice the molecular weight of the CNTN6 protein on western blot analysis (Supplementary Figure 8). However, further molecular studies would be required to firmly establish impaired ligand interactions or protein folding.

### Clinical characterization of patients carrying CNTN5 or CNTN6 variants

CNTN5 and CNTN6 are interesting for ASD because of their role in the development of sensory-motor neuronal pathways.^28, 29, 30, 31^ We therefore explored sensory-motor abnormalities in the patients carrying CNTN5 or CNTN6 variants.

**Figure 4:**
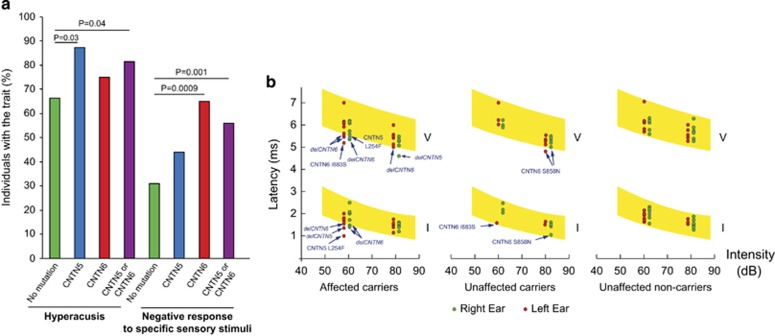
Clinical investigation and auditory brainstem responses in CNTN5 or CNTN6 rare variant carriers and non-carriers. (**a**) Individuals with ASD carrying CNTN5 (*N*=24) or CNTN6 (*N*=24) rare variants are more prone to suffer from hyperacusis or to display abnormal idiosyncratic-negative response to specific sensory stimuli than the rest of the cohort of patients with ASD (*N*=544). (**b**) Auditory brain response in the probands carrying *CNTN5* or *CNTN6* rare variants, and their first-degree relatives. The graph represents response latencies (in milliseconds) for waves I, III and V as a function of signal intensity (in decibels) in right ear (green dots) and left ear (red dots). Normal ranges of response (5–95th percentile) are shown in yellow (based on manufacturer information); variants of individuals outside the normal range are indicated. Electrodes were placed at Fz, ipsilateral earlobe and contralateral earlobe (ground). Responses were amplified with a gain of 100 000 and digitally filtered with a bandwidth of 100–3000 Hz. Each ear was stimulated by alternating 60 and 80 dB clicks with a presentation rate of 29 per second. Clicks were delivered using Etymotic Research ER-3 insert earphones. Two separate recordings that included 2000 individual sweeps were averaged. ASD, autism spectrum disorder.

Based on the ADI-R and clinical evaluation, the vast majority of the patients carrying a CNTN5 or CNTN6 variant presented with fine or gross motor coordination problems, but did not statistically differ from the overall cohort for these symptoms (*P*=0.44). When considering the item of the ADI-R related to excessive sensibility to noise (Figure 4), we found that probands carrying CNTN5 or CNTN6 variants were more prone to suffer from hyperacusis (39/48; 81%) than the rest of the cohort (360/548; 66% *P*=0.036). They also displayed more abnormal idiosyncratic-negative response to specific sensory stimuli (23/41; 56%) than the rest of the cohort (153/505; 30% *P*=0.001).

We then ascertained ABR for a subset of families with CNTN variants that were re-evaluated on this purpose (Figure 4). A total of 24 individuals (8 probands with ASD, 2 siblings with ASD, 2 fathers with ASD and 12 unaffected relatives) from 8 independent families were enrolled in this study. Three probands carried a *CNTN6* deletion, three carried a *CNTN6*-coding sequence variant affecting neurite outgrowth (CNTN6^G310S^, CNTN6^I683S^ and CNTN6^S858N^), one carried a *CNTN5* deletion and one carried a CNTN5^L254F^ variant predicted as deleterious. The clinical description of the probands is presented in Supplementary Table 8. Remarkably, except the carrier of CNTN6^S858N^, all probands suffered from hyperacusis (which was painful during ear examination). None of the individuals without variants and none the unaffected relatives carrying variant showed over sensitivity to sound. ABRs were recorded for intensities of 60 and 80 dB and frequencies of 29 clicks per second. Wave latencies tended to be shorter in subjects carrying a CNTN5 or CNTN6 variant compared with non-carriers (Figure 4 and Supplementary Table 9).

## Discussion

In the present study, we identified *CNTN5* and *CNTN6* rare variants in individuals with ASD and investigated their impact on clinical phenotypes. Although we observed *CNTN5* genetic abnormalities in patients with ASD, the demonstration of an association between this gene and neuropsychiatric disorder would require larger cohorts of patients. In contrast, we provide further support that *CNTN6* mutations are risk factors for ASD. Our results confirm data from previous reports describing patients diagnosed with ASD and/or ID carrying inherited or *de novo CNTN6* CNVs or SNVs.^49, 50, 51, 52^

Interestingly, two previous studies from Van Daalen *et al.*^49^ and Hu *et al.*^52^ reported that relatives of patients carrying *CNTN5* or *CNTN6* CNVs were diagnosed with neuropsychiatric disorders or had deficits in social interactions.^49, 52^ Similarly, in our study, several parents carrying *CNTN6* variants were diagnosed with ASD. This co-segregation between *CNTN6* variants and the presence of neuropsychiatric disorders in the relatives could explain why Murdoch *et al.*^63^ did not found a significant association between *CNTN6* rare variants and ASD in the Simons Simplex Collection. Indeed, having first-degree relatives on the autism spectrum is an exclusion criterion of the Simons Simplex Collection.^75^

We, and others,^49, 50, 51, 52^ showed that CNTN6 mutations are not fully penetrant, but they might represent risk factors for ASD in specific genetic backgrounds.^76^ Using additional genotyping and sequencing data, we could detect multiple hits in the patients carrying the CNTN6 variants. Several mutations were affecting genes such as *GRID2, DMD* and *RAB39B* that are known to be risk factors for ID, neuromuscular disorder, epilepsy and in some cases ASD.^73, 77, 78, 79, 80^ It would now be interesting to ascertain if patients with some genetic backgrounds are more sensitive to *CNTN6* mutations. Testing for such association would, however, require very large cohorts of patients and controls with genetic and phenotypic data.

Given the expression patterns and the roles of CNTN5 and CNTN6 in the development of sensory-motor neuronal pathways, either loss or gain of neurite outgrowth might perturb the sensory-motor functions of patients with ASD. CNTN6 is highly expressed in the granule cells of the cerebellum (but not in Purkinje cells) and is involved in the postnatal synapse formation. This expression pattern in the cerebellum is similar to that of SHANK3, another gene associated with ASD.^23, 81^ Both CNTN5 and CNTN6 are also expressed in the inferior colliculus, the principal midbrain nucleus of the auditory pathway.^32, 34^ This structure receives input from several peripheral brainstem nuclei in the auditory pathway, as well as from the auditory cortex. Remarkably, mice lacking CNTN5 display disorganized tonotopy and auditory brain response abnormalities.^82^ Accordingly, we observed that almost all the patients who we reassessed suffered from painful hypersensitivity to noise. Some of these patients also displayed shortened waves latencies in their ABRs. Thus, abnormal gene dosage of *CNTN5* or *CNTN6* might represent a risk factor for the auditory problems recurrently reported in patients with ASD.^9, 10, 11, 12, 13, 14, 15, 16^

In conclusion, we identified rare *CNTN5* and *CNTN6* deleterious mutations in a subset of individuals with ASD. Both CNTN5 and CNTN6 are expressed at high levels in the auditory pathway. This is in accordance with the fact that a majority of patients with mutations displayed painful hyperacusis. There is an emerging literature on the prominent role of sensory dysfunctions in the development of ASD. A better understanding of the genetic pathways associated with these abnormalities should lead to better clinical interventions in ASD.

## Figures and Tables

**Table 1:** Multiple hits identified in patients with ASD carrying CNTN6 rare variants

*IID*	*Status*	*Sex*	*Cohort*	*CNTN6 variant*	*Maternally inherited variants*	*Paternally inherited variants*
AU-RD-LAB-192-003	ASD	M	FR	**CNTN6 chr3:1336137-1368840 32 kb deletion (MI)**		**NPHP1-chr2:110863095-111148771 285 kb duplication**
AU-FRA-MIC-033-003	ASD	M	FR	CNTN6-I683S (PI)		CTTNBP2-P1586L, PTPRM-S706G
C0733-011-017-005	ASD	M	FR	CNTN6-P770L (*de novo*)		**GRID2-chr4:92606953-93480717 873 kb duplication,** NINL-R112Q, CADPS2-S11L, ATP2B4-V543I
AU-RD-BOR-104-003	ASD	M	FR	CNTN6-S419C (PI)	TAF1L-I1335T, RB1CC1-Y513H	ANK2-E1449G
AU-RD-BUR-237-004^a^	ASD	M	FR	CNTN6-S858N (PI)		**PARK2-chr6:162614973-162791579 176 kb deletion**
AU-RD-BUR-237-005^a^	ASD	M	FR	CNTN6-S858N (PI)		**PARK2-chr6:162614973-162791579 176 kb deletion**
AU-RD-BOU-233-005	ASD	M	FR	CNTN6-T958I (MI)	HDAC4-A786T	MYO1A-G651R, GRIK5-R582H
1-0232-003/5241-3	ASD	M	CA	**CNTN6-chr3:3:464181-1251877 787 kb duplication (PI)**	**DMD-chrX:31883357-31912783 29 kb deletion**, DISC1-E783Q, POGZ-P955R, JMJD1C-E2531K, CACNA1C-G37R, GNPTAB-N351S, AKAP9-V3780L, CREBBP-L551I	COG1-Q301H, NRCAM-G1168V, TGFBR2-S578T, ARFGEF2-G1298S, LRP2-R3888H, RELN-G370R, SPTAN1-E298G, ;MYO1A-R628G, DAPK1-G1348V, CREBBP-L551I
2-1335-003	ASD	M	CA	CNTN6-G310S (MI)	***USP45*****-splice-A99930627G**, CACNA1D-A1305T, SUCLG2-K101R, GTF2I-N440S, CEP290-D433G	**chr15:29322000-29584000 262 kb duplication (NDNL2 and APBA2)**, KMT2C-C1114R, CNTNAP4-A600T, SYNE1-L3050V, LRP2-G3470D, NPHP1-S629L, CTTNBP2-E9K, MFRP-L458F, GTF2I-N440S, CEP290-D433G
2-1335-004	ASD	M	CA	CNTN6-G310S (MI)	CNTNAP2-C9S, MYO1A-K485E, CACNA1H-F1452S, HDLBP-P372A, (CEP290-D433G Inheritance unknown)	**KAT6B-chr10:76755251-76841351 86 kb duplication**, CNTNAP4-A600T, RELN-E2174K, (CEP290-D433G Inheritance unknown)
2-0018-003	ASD	M	CA	**CNTN6-W923X (MI)**	**RAB39B-E187X**, DNAH10-E3125K, DMD-M264T, PGAP3-A141G	CD163L1-T1307A
2-0018-004	ASD	M	CA	**CNTN6-W923X (MI)**	**RAB39B-E187X**, DNAH10-E3125K, CDH9-E603G, PGAP3-A141G, JMJD1C-E2531K	PIGV-L457F, RAI1-A381V, USP45-C62F, KIAA1033-R717Q, PAH-T380M, ASXL3-L2067R
2-1380-proband	ASD	M	CA	CNTN6-I529L (PI)	**CHST5-I121fs,** CADPS2-V799L, FBXO40-G517E, ASPM-E216K, GNPTAB-R46Q,	**HDAC4-M902fs**, **NDUFA5-Y3X**, MFRP-I331T, ARID1A-R1323C, PAH-I421T, CACNA1G-V96M, GFAP-D157N, SPARCL1-T363S, MYO1A-D67H
1-0366-006	ASD	M	CA	CNTN6-I529L (MI)	***KLC1*****-splice-G104145883T**, CNTN3-V950I, COL4A1-G936A,	CNTN3-T901M, TTC8-K95R, ERBB2IP-H1045Y
2-1222-proband	ASD	M	CA	CNTN6-I109V (MI)	***CD44*****-splice-G35243921A**, ***MIR17HG*****-splice-T92005592G**, ZBTB20-D315E, TAF1L-E935K, ASTN2-A154V, LRP2-R2126G	**FCRL6-Q276X**, CNTNAP4-I763F, ETFB-R98C, KHDRBS2-Y305C, KIAA1033-T1046I, ANKRD11-R840Q
2-1357-003	ASD	M	CA	CNTN6-S995T (PI)	**FGFR2-X681W**, COL4A1-P304L, MYO9B-V1702I, FKRP-N480I, ERCC5-R1302W, ZNF423-L1068M, ASMT-T235N, HDAC4-P428T, AP4B1-I397V, CHD7-S2490P,	***DHCR7*****-splice-C71146886G**, ***RAD21*****-splice-C117879001T,** C5orf42-G3098R
2-1195-proband	ASD	M	CA	CNTN6-A827V (PI)	PARK2-Q34R, LMX1B-G210R, DLGAP2-R566H, PCNT-P2420L	CNTN3-H625R, CNTN3-H75D, COG1-Y744C, TTC8-G411R, SNTG2-Y212C, HOXA1-R121S, FGFR2-I8S, PRSS12-V424I, CDH15-L240F
1-0273-004	ASD	M	CA	CNTN6-P38A (PI)	**KATNAL2-Q53X**, ERBB2IP-D1135N, IQSEC2-P899L, DAG1-E37Q, PIK3CG-S1003G, ANKRD11-K2038E, ARFGEF2-E731K, BRWD1-D675V, BZRAP1-P1073A, ERCC6-G1322V, ST3GAL3-R414Q, PVRL1-G44S, SYNE1-Y7418C	***FOLR1*****-splice-T71906793C**, **SRCAP-T1156fs**, CNTNAP1-R1200C, DIAPH3-P596, RARS2-R560C, ERCC2-V476I, VPS13B-T3012S, PIGV-P426L
1-0366-003	ASD	M	CA	CNTN6-I529L (MI)	***KLC1*****-splice-G104145883T**, ***ASMT*****-splice-T1748834C**, COL4A1-G936A, RANBP17-R491H, GFAP-E312Q	CEP41-P206A, TTC8-K95R, CARKD-V208L, AFF4-R225C
1-0518-proband	ASD	M	CA	CNTN6-N334T (MI)	***MIR17HG*****-splice-T92004837C**, CNTN3-T657M, TTC8-M240I, PMM2-R141H, PTK7-S136L	***SOX5*****-splice-C24521496-**, NINL-Q522H, DLGAP2-R785C, GRID2-F974Y, ATP2B4-E661K, CEP290-D1413H

Abbreviations: ASD, autism spectrum disorder; CA, Canada; FR, France; M, male; MAF, minor allele frequency; MI, maternally inherited; PI, paternally inherited. When both parents are carriers of the variant, the inheritance is indicated as unknown.

For CNVs, only the rare and exonic CNVs are indicated. For SNVs, only the rare variants (MAF<1% in 1000 genomes and ExAC) and considered deleterious by at least 2 algorithms (CADD Phred score≥20; SIFT≤0.05, PolyPhen2≥0.453, Mutation Assessor≥2, vertebrate PhyloP≥2) are indicated. The complete list of ASD-risk genes is available in Supplementary Table 10.

a Individuals with no available WES/WGS information. Likely gene disrupting mutations are indicated in bold.

## Supplementary Material

Supplementary Informations
