# Supplementary material for: CNTN6 mutations are risk factors for abnormal auditory sensory perception in autism spectrum disorders
Source: Mol Psychiatry. 2016 May 10;22(4):625–33. doi: 10.1038/mp.2016.61 (PMC5378808; doi:10.1038/mp.2016.61)
Supplement: Supplementary Informations [file mp201661x1.doc]

**CNTN6 mutations are risk factors for abnormal auditory**

**sensory perception in autism spectrum disorders**

Mercati O^1,2,3, $^, Huguet G^1,2,3,$^, Danckaert A^4^, André-Leroux G^5,6^, Maruani A^7^, Bellinzoni M^5^, Rolland T^1,2,3^,, Gouder L^1,2,3^, Mathieu A^1,2,3^, Buratti J^1,2,3^, Amsellem F^7^, Benabou M^1,2,3^, Van-Gils J^1,2,3^, Beggiato A^7^, Konyukh M^1,2,3^, Bourgeois J-P^1,2,3^, Gazzellone MJ^8^, Yuen RKC^8^, Walker S^8^, Delépine M^9^, Boland A^9^, Régnault B^10^, Francois M^11^, Van Den Abbeele T^11^, Mosca-Boidron AL^12^, Faivre L^12^, Shimoda Y^13^, Watanabe K^13^, Bonneau D^14^, Rastam M^15,16^, Leboyer M^17,18,19,20^, Scherer SW^8, 21^, Gillberg C^16^, Delorme R^1,2,3,7^, Cloëz-Tayarani I*^1,2,3^, Bourgeron T*^1,2,3,16,20^

**Supplementary data**

**Supplementary Table S1.** Description of the patients from the PARIS cohort used for the CNV screening

**Supplementary Table S2.** Description of the cohorts used for CNV screening

**Supplementary Table S3.** Description of the patients from the PARIS cohort used for the SNV screening

**Supplementary Table S4.** Primers used for mutation screening of CNTN5 and CNTN6

**Supplementary Table S5.** Primers used for site-directed mutagenesis of CNTN5 and CNTN6 variants

**Supplementary Table S6.** CNTN5 coding variants identified in this study

**Supplementary Table S7.** CNTN6 coding variants identified in this study

**Supplementary Table S8.** Clinical characterization of the patients carrying CNTN5 or CNTN6 variants and evaluated for auditory brainstem responses

**Supplementary Table S9.** Auditory brainstem responses of the patients and the controls.

**Supplementary Figure S1.** Genetic and clinical characterizations of family AUDIJ001 and AUDIJ002 carrying CNTN5 CNVs

**Supplementary Figure S2.** CNTN5 and CNTN6 CNVs identified in patients with ASD

**Supplementary Figure S3.** Localization of the exonic CNVs affecting CNTN5 or CNTN6 in patients and controls

**Supplementary Figure S4.** Families with CNTN5 and CNTN6 coding SNVs from the PARIS cohort

**Supplementary Figure S5.** Families with CNTN5 and CNTN6 coding SNVs from Canada

**Supplementary Figure S6**. Frequency of CNTN5 and CNTN6 private variants in this study, in Murdoch et al. PLOS Genetics (2015) and in the ExAC cohort

**Supplementary Figure S7. Venn diagram of the ASD-risk genes from different databases.**

**Supplementary Figure S8. Impact of the CNTN6 variants on neurite outgrowth.**

**Supplementary Figure S9. Effect of CNTN6 variants on protein structure.**

**Supplementary Table S1. Description of the patients from the PARIS cohort used for CNV screening.**

|  | **Total** | **Sex** | | | **Age class** | | | | **Array platform (Illumina)** | | | |
| --- | --- | --- | --- | --- | --- | --- | --- | --- | --- | --- | --- | --- |
|  |  | **Male** | **Female** |  | **< 12 y** | **12 – <18 y** | ≥ **18y** | **NA** | **660W Quad** | **1M Duo – 1M** | **Omni1** | **Omni2.5** |
| ASD with ID | n = 361 | 294 | 67 |  | 4 | 68 | 203 | 86 | 0 | 250 | 108 | 3 |
| ASD without ID | n = 195 | 162 | 33 |  | 3 | 43 | 113 | 36 | 27 | 151 | 14 | 3 |
| NA | n = 77 | 66 | 11 |  | 4 | 8 | 1 | 64 | 0 | 36 | 41 | 0 |
| **Total** | **n = 633** | **522** | **111** |  | **11** | **119** | **317** | **186** | **27** | **437** | **163** | **6** |

**Supplementary Table S2.** Description of the cohorts used for CNV screening.

| **Project** | **Technologies** | **N individuals** | **CNTN5** | | **CNTN6** | |
| --- | --- | --- | --- | --- | --- | --- |
|  |  |  | **Loss** | **Gain** | **Loss** | **Gain** |
| **Patients with ASD**  The PARIS cohort  Pinto et al. 2010  **Total Patients with ASD**  **Controls ^a^**  This study | Illumina (1M, 1M duo, Omni 1, 2.5M, 5M)  Illumina 1M  **Illumina**  Illumina (660Wq, 1M, 1M duo, Omni 1, 5M) | 633  901  **1534**  **2126** | 1  0  **1**  0 | 0  0  **0**  0 | 4  2  **6**  0 | 0  2  **2**  1 |
| SAGE+Hapmap3 (Pinto et al. 2010) | Illumina 1M | 1287 | 0 | 1 | 0 | 5 |
| HBAC | Illumina 1M duo | 2566 | 1 | 1 | 0 | 4 |
| KORA | Illumina 2.5M Quad | 1775 | 0 | 1 | 1 | 2 |
| COGEND | Illumina 2.5M | 1182 | 0 | 0 | 0 | 0 |
| **Total Controls** | **Illumina** | **8936** | **1** | **3** | **1** | **12** |

^a^ Control datasets were obtained, along with permission for use, from the database of Genotypes and Phenotypes (dbGaP) found at http://www-ncbi-nlm-nih-gov.myaccess.library.utoronto.ca/gap through accession numbers phs000169.v1.p1 (Whole Genome Association Study of Visceral Adiposity in the HABC Study), phs000303.v1.p1 (Genetic Epidemiology of Refractive Error in the KORA Study), and phs000404.v1.p1 (COGEND; The Genetic Architecture of Smoking and Smoking Cessation).

**Supplementary Table S3.** Description of the patients from the PARIS cohort used for SNV screening.

|  | **Total** | **Sex** | | | **Age class** | | | | | **Family status** | | |
| --- | --- | --- | --- | --- | --- | --- | --- | --- | --- | --- | --- | --- |
|  |  | **Male** | **Female** |  | | **< 12 y** | **12 – <18 y** | ≥ **18y** | **NA** | **simplex** | **multiplex** | **single** |
| ASD with ID | n = 125 | 95 | 30 |  | | 1 | 22 | 93 | 9 | 89 | 36 | 0 |
| ASD without ID | n = 82 | 66 | 16 |  | | 2 | 14 | 60 | 6 | 46 | 20 | 16 |
| NA | n = 5 | 5 | 0 |  | | 1 | 0 | 4 | 0 | 3 | 2 | 0 |
| **Total** | **n =212** | **166** | **46** |  | | **4** | **36** | **157** | **15** | **138** | **58** | **16** |

**Table S4. Primers used for mutation screening of CNTN5 and CNTN6**

| **Exon** | **Size (bp)** | **F primer** | **Forward primer sequence (5'-3')** | **R primer** | **Reverse primer sequence (5'-3')** | **Tm (°C)** |
| --- | --- | --- | --- | --- | --- | --- |
| 3 | 418 | CNTN5_1 | TCTATCTCAAACAGGGCACTC | CNTN5_2 | GCCTTCTGAAAGCTAGATGG | 60 |
| 4 | 524 | CNTN5_3 | TGCAGCTCCAAAGAAGGTTT | CNTN5_4 | TCTCCCCTAACATCCTCCAG | 60 |
| 5 | 763 | CNTN5_5 | CACCCCAGAATTGTTAGCAC | CNTN5_6 | TTCCATTTCGAAGCCATCTT | 60 |
| 6 | 817 | CNTN5_7 | AGTTCGTGGCAATCCAGTTC | CNTN5_8 | CATGAAAAGGACTTCAACCTG | 60 |
| 7 | 731 | CNTN5_9 | GAGAGAATGGGCGTTATTGG | CNTN5_10 | CGCTTCCTTTGTGCTCTAGG | 60 |
| 8 | 527 | CNTN5_11 | GGCTTTGTTGTTTGAGCTTT | CNTN5_12 | TTCTCCCTTTCTGGCATTAG | 60 |
| 9 | 396 | CNTN5_13 | TCCTCAGGGGAAGACTAGGA | CNTN5_14 | GGGGAAATGACACCTAGCAA | 60 |
| 10 | 549 | CNTN5_15 | AACCCACGTGGAAAGTGAAA | CNTN5_16 | CACCGATAATTCCCCATTCA | 60 |
| 11 | 556 | CNTN5_17 | CCCCACTACATTGAAAGAGC | CNTN5_18 | GCCTAGTTTGTCCTGTGTGA | 60 |
| 12 | 591 | CNTN5_19 | TACATTGCTAAGTGGCCAAA | CNTN5_20 | CTACCCTCAAGGCATTCTTC | 60 |
| 13 | 621 | CNTN5_21 | AGGTCACCTCCATCTTACCA | CNTN5_22 | TACGTCTCACGGTACTGCAA | 60 |
| 14 | 620 | CNTN5_23 | AAACTCAGGGTCACCCCTTC | CNTN5_24 | TGCCAATAAGAGGTTCCTTCA | 60 |
| 15 | 850 | CNTN5_25 | AGGAACAGAGGCTCTTAAATC | CNTN5_26 | GGCTAATGTGCATTTATGACT | 56 |
| 16 | 593 | CNTN5_27 | CCCACAAGTTGCTGTTCCTT | CNTN5_28 | GATGTGCTCAAAGTCCATGC | 60 |
| 17 | 636 | CNTN5_29 | TTTCCCCCAGAAATCCTAGA | CNTN5_30 | GAGTGGGTCCACAGGAACTC | 60 |
| 18 | 766 | CNTN5_31 | GGAGGTGACACCATGACCAC | CNTN5_32 | TAGGTCAAGGAAGGGCAATG | 60 |
| 19 | 839 | CNTN5_33 | CATGGTTTGGTGCAATGAAG | CNTN5_34 | TCCTCCCACTGTTTTTAGCC | 60 |
| 20 | 849 | CNTN5_35 | TTTCCCCAACATGTTAATCC | CNTN5_36 | CCAGAGAAAGGAATACACTGC | 56 |
| 21 | 649 | CNTN5_37 | TTCCCCAGAAATGTCTTAGC | CNTN5_38 | ACCTGTAGCTCCTGCTTTTG | 60 |
| 22 | 509 | CNTN5_39 | CTGCTGTAGTGCTGCAATTT | CNTN5_40 | TCAAAACTCCCCTTTGACTC | 60 |
| 23 | 515 | CNTN5_47 | TGCCAGCCAACTACTAAGCA | CNTN5_48 | GCCTAAATTCTGCTGGTCCT | 60 |
| 24 | 522 | CNTN5_43 | TGGCCCTTTTGTGTACTTCT | CNTN5_44 | TCTGCAATTGTGAGCAGATT | 60 |
| 25 | 702 | CNTN5_45 | TCTCATGATTGGATGTGTTTG | CNTN5_46 | CTTAACAAATGGATGGCACA | 60 |
| 2 | 646 | CNTN6_1 | ATTATGATGCAACCCCCTTA | CNTN6_2 | AAACTTTCCCTGTCTGAGTCAT | 60 |
| 3 | 460 | CNTN6_3 | GGGTAATAGGGGATTGGTTT | CNTN6_4 | ATTGTCACATGGCTGCATAC | 60 |
| 4 | 469 | CNTN6_5 | CCAAGACAGCTACCTTTGGT | CNTN6_6 | CATTGTCAAAAGCCCATAAA | 60 |
| 5 | 542 | CNTN6_7 | ACCTTCCTTGGACAGACAAA | CNTN6_8 | GCACTTGACGAGAGTCACAA | 60 |
| 6 | 625 | CNTN6_9 | AAAAGTCAATGATGGGGAAA | CNTN6_10 | GAACCATGTCTACTGCACCA | 60 |
| 7 | 602 | CNTN6_11 | TTAGCTGAAAACATCCCGTTG | CNTN6_12 | GAATTGCAGCCCTGATCATT | 60 |
| 8 | 486 | CNTN6_13 | GCATACGGCAGGCACTTAAT | CNTN6_14 | TCCCGTGACACTTTTTCACA | 60 |
| 9 | 591 | CNTN6_15 | ATTGAAGCTCCTGCATGTCC | CNTN6_16 | TATCCGTGTCGATGATGACG | 60 |
| 10 | 589 | CNTN6_17 | GAAAGCCAAGGCAGTGTTCT | CNTN6_18 | AAAATTGGGTTGGGATTATGC | 60 |
| 11 | 591 | CNTN6_19 | GGTCCAAGAACACCTTGAGC | CNTN6_20 | TTCAGGAGGAAGCCTTCAAA | 60 |
| 12 | 559 | CNTN6_21 | TGCTTTGAAATCGACAATGA | CNTN6_22 | ACTGGCAAAGTGTGCATGTT | 60 |
| 13 | 468 | CNTN6_23 | TGTAGAACTCCAGGTTGCAT | CNTN6_24 | TCTACGGAAGACACGAAACA | 60 |
| 14 | 492 | CNTN6_25 | TTTGTTTCGTGTCTTCCGTA | CNTN6_26 | TACCCATACCATGGAGTCCT | 60 |
| 15 | 533 | CNTN6_27 | TGCAATCATCCTATGCCTGA | CNTN6_28 | AAGGCACCTAGGTTGGGACT | 60 |
| 16 | 587 | CNTN6_29 | CAGGCTGTTGCTACAGGTGA | CNTN6_30 | GATGCGATGCAAACACAGTT | 60 |
| 17 | 496 | CNTN6_31 | GAGCCAGGGTGCTCATGTAT | CNTN6_32 | TTGGGAGAAAATTGTGCTGA | 60 |
| 18+19 | 780 | CNTN6_33 | TGGCATTCCAAATAAAAGTG | CNTN6_34 | GCTCACCTACCCTGTCTCAA | 60 |
| 20 | 602 | CNTN6_35 | CCTTGTGGTTGTGGTTGATA | CNTN6_36 | TACCACCTAACGTTCCAAGG | 60 |
| 21 | 590 | CNTN6_37 | CATTCCCAAATGACCAGATT | CNTN6_38 | TGCACATAAGCCATCATCTT | 60 |
| 22 | 590 | CNTN6_39 | CAGTGACTTTCCACAGCAAG | CNTN6_40 | CTCATCACCTTTTGCTCACA | 60 |
| 23 | 573 | CNTN6_41 | GACACGGTAAGGCACTTTCT | CNTN6_42 | AGCATCATTGAGGGGAGTTA | 60 |

PCR reactions were performed using BioTaq DNA polymerase (Gentaur) and the following protocol: 94°C for 4 min, followed by 35 cycles of: 94°C-initiation for 30 seconds; annealing for 40 seconds and 72°C-elongation for 30 seconds; and a final 72°C-elongation for 10 min.

**Table S5. Primers used for site-directed mutagenesis of CNTN6** variants

| **a.a. change** | **rat cDNA nt change(s)** | **Forward primer (5'-3')** | **Reverse primer (5'-3')** |
| --- | --- | --- | --- |
| CNTN6_S57L | c170t + t171a | AGTTGTACTGCAAGTGGATACCCTT**TA**CCCCACTACAGGT | ACCTGTAGTGGGG**TA**AAGGGTATCCACTTGCAGTACAACT |
| CNTN6_R303Q | g908a | CGTTGCAGGTAACCTTC**A**AGGAAGAAACCTTGCAA | TTGCAAGGTTTCTTCCT**T**GAAGGTTACCTGCAACG |
| CNTN6_G310S | g928a | GAGGAAGAAACCTTGCAAAG**A**GTCAACTCATTTTCTATGCT | AGCATAGAAAATGAGTTGAC**T**CTTTGCAAGGTTTCTTCCTC |
| CNTN6_N377S | a1130g | gactcttatcatcaccatgctga**g**cgtgtcagactc | gagtctgacacg**c**tcagcatggtgatgataagagtc |
| CNTN6_S419C | c1256g + a1257t | TCCAGATTTCTCAAAAAATCCCATTAAAAAAATTT**GT**GTTGTTCAAGTTGGTGGTG | CACCACCAACTTGAACAAC**AC**AAATTTTTTTAATGGGATTTTTTGAGAAATCTGGA |
| CNTN6_G678S | g2032t + g2033c | AATATGAATTTCGTGTTGTTGCT**TC**GAACAACATTGGAATTGGAGAGC | GCTCTCCAATTCCAATGTTGTTC**GA**AGCAACAACACGAAATTCATATT |
| CNTN6_I683S | t2048g | TGTTGCTGGGAACAACATTGGAA**G**TGGAGAGCCAAG | CTTGGCTCTCCA**C**TTCCAATGTTGTTCCCAGCAACA |
| CNTN6_P770S | c2308t | ACAGAAATGAAAGCATCATG**T**CTTTGTCTCCCTTTGAAGTC | GACTTCAAAGGGAGACAAAG**A**CATGATGCTTTCATTTCTGT |
| CNTN6_S858N | g2573a | AAAGAATCTATGATTGGGAAAATTAGAGTCA**A**TGGAAATGTCACAACC | GGTTGTGACATTTCCA**T**TGACTCTAATTTTCCCAATCATAGATTCTTT |
| CNTN6_T958I | c2873t | GTAAAACTCATGTCTTGGAAACAAATAATA**T**ATCAGCTGAGCTACTG | CAGTAGCTCAGCTGAT**A**TATTATTTGTTTCCAAGACATGAGTTTTAC |

Mutated nucleotides are shown in blue.

**Supplementary Table S6. CNTN5 coding variants identified in this study**

|  | Detected variants | | | | Frequency ^c^ | | | Domain | PolyPhen2 (Hum_Div) ^d^ | Inheritance | |
| --- | --- | --- | --- | --- | --- | --- | --- | --- | --- | --- | --- |
|  | Exon^a^ | Nucleotide change ^b^  (chr. 11) | dbSNP  (build 137) | a. a. change | ASD PARIS ^c^  (n=212) | ASD Canada  (n=289) | Controls  (n=217) |  |  | PARIS | Canada |
| ASD only | 8 | g. 99827588 G>T  ^#^ | unknown | p. D242Y | 1 | 0 | 0 | Ig2 | deleterious | M | - |
|  | 8 | g. 99827624 C>T ^#^ | unknown | p. L254F | 1 | 0 | 0 | Ig2 | deleterious | P | - |
|  | 10 | g. 99931978 G>A | rs200767659 | p. G339S | 0 | 1 | 0 | Ig3 | deleterious | - | P |
|  | 10 | g. 99932054 C>T | rs200797524 | p. A364V | 1 | 0 | 0 | Ig3 | deleterious | M | - |
|  | 14 | g. 100061860 T>C | unknown | p. I528T | 1 | 0 | 0 | Ig5 | neutral | M | - |
|  | 20 | g. 100169923 A>C | rs200512456 | p. E805D | 0 | 2 | 0 | FN2 | deleterious | - | 2 M |
|  | 20 | g. 100170018 G>A | rs199937129 | p. R837Q | 0 | 1 | 0 | FN2 | deleterious | - | M |
|  | 21 | g. 100179121 C>T | rs200726875 | p. A884V | 0 | 1 | 0 | FN3 | deleterious | - | P |
|  | 21 | g. 100179166 T>C | rs201978001 | p. I899T | 1 | 0 | 0 | FN3 | neutral | M | - |
|  | 21 | g. 100211262 C>T | rs201747311 | p. S933F | 0 | 1 | 0 | FN3 | deleterious | - | 1 M |
|  | 21 | g. 100211267 G>A | unknown | p. V935I | 0 | 1 | 0 | FN3 | neutral | - | M |
| ASD and controls | 6 | g. 99715891 T>G | rs61749255 | p. I158M | 1 | 6 | 2 | Ig1 | deleterious | - | 2 P, 4 M |
|  | 14 | g. 100061865 A>G | rs11223168 | p. I530V | 32 htz | 27 htz (3 hmz) | 40 htz (1 hmz) | Ig5 | neutral | - | - |
|  | 19 | g. 100168410 T>A | rs141228828 | p. L790I | 6 | 2 | 4 | FN2 | deleterious | 4 P, 1 M | 1 M |
|  | 20 | g. 100170119 G>C | rs200130506 | p. A871P | 1 | 0 | 1 | - | deleterious | - | - |
|  | 25 | g. 100226883 T>A | rs1216183 | p. S1079T | 50 htz (2 hmz) | 54 htz (4 hmz) | 46 htz (6 hmz) | - | neutral | - | - |
|  | 24 | g. 100221596 A>T | rs1944169 | p. Y1065F | 0 | 0^c^ | 1 | - | neutral | M | - |
| Controls only | 8 | g. 99827639 G>A | unknown | p. V259I | 0 | 0 | 1 | Ig2 | neutral | - | - |
|  | 10 | g. 99931945 C>G  ^#^ | rs201910584 | p. P328A | 0 | 0 | 1 | Ig3 | deleterious | - | - |
|  | 10 | g. 99932033 C>T ^#^ | rs56122118 | p. P357L | 0 | 0 | 1 | Ig3 | deleterious | - | - |
|  | 18 | g. 100141888 delA | unknown | p. E743Dfs*5 | 0 | 0 | 1 | FN1 | - | - | - |

^a^ Variants in Exon 4 of CNTN5 were not reported since there were discrepancies between results obtained through next generation sequencing and Sanger sequencing. ^b^Nucleotide positions correspond to the NCBI37/hg19 build. ^c^ In order to have geographically matched patients and controls, among the 226 patients with ASD, 14 individuals were non from European descent and were not included in the statistical analysis. Yet, one coding-sequence variant, CNTN5^Y1065F^, was identified in a patient from African origin. ^d^ PolyPhen2 ([*http://genetics.bwh.harvard.edu/pph2/*](http://genetics.bwh.harvard.edu/pph2/)), which classifies variants into benign, possibly damaging and probably damaging. htz: Heterozygote; hmz: homozygote. P : Paternal; M: Maternal

**Supplementary Table S7. CNTN6 coding variants identified in this study**

|  | Detected variants | | | | Frequency | | | Domain | PolyPhen2 (Hum_Div) ^b^ | Inheritance | |
| --- | --- | --- | --- | --- | --- | --- | --- | --- | --- | --- | --- |
|  | Exon | Nucleotide change ^a^  (chr. 11) | dbSNP  (build 137) | a. a. change | ASD PARIS  (n=212) | ASD Canada  (n=289) | Controls  (n=217) |  |  | PARIS | Canada |
| ASD only | 3 | g. 1262427 C>G | unknown | p. P38A | 0 | 1 | 0 | Ig1 | deleterious | - | P |
|  | 3 | g. 1262485 C>T | unknown | p. S57L | 1 | 0 | 0 | Ig1 | neutral | P | - |
|  | 4 | g. 1269644 A>G | unknown | p. I109V | 0 | 1 | 0 | Ig1 | neutral | - | M |
|  | 5 | g. 1320187 T>C | rs6808056 | p. F150S | 1 | 0 | 0 | Ig2 | neutral | P | - |
|  | 8 | g. 1363500 G>A | rs149799168 | p. G310S | 1 | 1 | 0 | - | deleterious | P | 1 M |
|  | 9 | g. 1367553 A>C | unknown | p. N334T | 0 | 1 | 0 | Ig4 | neutral | - | M |
|  | 11 | g. 1371511 C>G | unknown | p. S419C | 1 | 0 | 0 | Ig5 | deleterious | P | - |
|  | 13 | g. 1414075 A>C | rs139645898 | p. I529L | 0 | 2 | 0 | Ig6 | neutral | - | 1 P, 1 M |
|  | 16 | g. 1415710 T>G | unknown | p. I683S | 1 | 0 | 0 | FN1 | deleterious | P | - |
|  | 18 | g. 1424767 C>T | unknown | p. P770S | 1 | 0 | 0 | FN2 | deleterious | P | - |
|  | 18 | g. 1424768 C>T | unknown | p. P770L | 1 | 0 | 0 | FN2 | deleterious | de novo | - |
|  | 19 | g. 1425055 C>T | rs115667338 | p. A827V | 0 | 1 | 0 | FN3 | deleterious | - | P |
|  | 20 | g. 1427350 G>A | unknown | p. S858N | 1 | 0 | 0 | FN3 | neutral | P | - |
|  | 21 | g. 1443180 G>A | unknown | p. W923X | 0 | 1 | 0 | FN4 | - | - | 1 M |
|  | 22 | g. 1444057 C>T | unknown | p. T958I | 1 | 0 | 0 | FN4 | deleterious | M | - |
|  | 22 | g. 1444167 T>A | rs139391275 | p. S995T | 0 | 1 | 0 | FN4 | deleterious | - | P |
| ASD and Controls | 8 | g. 1363480 G>A | rs41293401 | p. R303Q | 3 htz (1 hmz) | 5 | 6 | Ig3 | deleterious | 1 M, 1 P | 4 P, 7 M |
| Controls only | 10 | g. 1369187 A>G | unknown | p. N377S | 0 | 0 | 1 | Ig4 | deleterious | - | - |
|  | 16 | g. 1415694 G>A | rs138682306 | p. G678S | 0 | 0 | 1 | FN1 | neutral | - | - |

**^a^** Nucleotide positions correspond to the NCBI37/hg19 build. ^b^ PolyPhen2 ([*http://genetics.bwh.harvard.edu/pph2/*](http://genetics.bwh.harvard.edu/pph2/)), which classifies variants into benign, possibly damaging and probably damaging . htz: Heterozygote; hmz: homozygote.

**Supplementary Table S8.** Clinical characterization of patients carrying CNTN5 or CNTN6 variants and evaluated for auditory brainstem responses

| **FAMILY** | AU-DIJ-001 | AU-DIJ-002 | AU-RD-124 | AU-FRA-035 | AU-RD-183 | AU-RD-192 | AU-FRA-033 | AU-RD-237 | AU-RD-068 |
| --- | --- | --- | --- | --- | --- | --- | --- | --- | --- |
| **GENETICS** |  |  |  |  |  |  |  |  |  |
| Gene | CNTN5 | CNTN5 | CNTN5 | CNTN6 | CNTN6 | CNTN6 | CNTN6 | CNTN6 | CNTN6 |
| Mutation | CNV/deletion | CNV/X5 | L254F | CNV/deletion | CNV/deletion | CNV/deletion | I683S | S858N | G310S |
| Transmission | unknown | maternal | paternal | paternal | paternal | maternal | paternal | paternal | paternal |
| **SEX** | f | f | m | f | m | m | m | m | m |
| **AGE** | 32 | 6 | 21 | 13 | 13 | 13 | 13 | 24 | 39 |
| **DIAGNOSIS ASSESSMENT** | ASD | ASD | ASD | ASD | ASD | ASD | ASD | ASD | ASD |
| **ADI-R** |  |  |  |  |  |  |  |  |  |
| Age at the time of evaluation (years) | 32 | 5 | 10 | 6 | 3 | 3 | 7 |  | 28 |
| Age of first symptoms (months) |  | 24 | 2 | 18 |  | 18 | 30 |  | 10 |
| Social domain (cut- off:10) | 13 | 9 | 28 | 23 | 13 | 28 | 17 |  | 21 |
| Communication domain: verbal (cut-off: 8) & non verbal (cut-off: 7) | 13 - 7 | 13 | 11 (nv) | 18 - 12 | 11 (nv) | 11 (nv) | 10 - 6 |  | 18 - 11 |
| Repetitive domain (cut-off: 3) | 4 | 1 | 5 | 4 | 4 | 4 | 8 |  | 9 |
|  |  |  |  |  |  |  |  |  |  |
| **ADOS** |  |  |  |  |  |  |  |  |  |
| Communication domain (cut-off: 5) |  | 2 | 6 | 9 |  | 7 | 4 |  | 10 |
| Social interaction domain (cut-off: 6) |  | 4 | 12 | 14 |  | 7 | 15 |  | 13 |
| Total |  | 6 | 18 | 23 |  | 14 | 19 |  | 23 |
| Interest and Behaviors |  |  | 2 | 1 |  |  | 2 |  | 1 |
|  |  |  |  |  |  |  |  |  |  |
| **DSM-5 criteria** | yes | yes | yes | yes | yes | yes | yes |  | yes |
| **IQ MEASUREMENT** |  |  |  |  |  |  |  |  |  |
| Scales | RPM | Wechsler | RPM | RPM | Wechsler | RPM | Wechsler | RPM | RPM |
| Full IQ scores | 70 | 82 | 75 | 84 | 96 | 93 | 85 | 84 | 66 |
| **MEDICAL HISTORY** |  |  |  |  |  |  |  |  |  |
| **Pregnancy and delivery** |  |  |  |  |  |  |  |  |  |
| Course of pregnancy | normal | normal | normal | normal | normal | normal | normal | twin | normal |
| Delivery course | eutocic | dystocic | dystocic | dystocic | eutocic | eutocic | dystocic | eutocic | eutocic |
| APGAR at 1 et 10 min (/10) |  | 10 - 10 | 9 - 10 | 9 - 10 | 10 - 10 | 10 - 10 | 8 - 10 | 10 - 10 | 10 - 10 |
| Birth heigh (cm) / weight (g) / head circumference (cm) |  | 46/2790/32 | 54/3780/36 | 49/2600/ -- | 51/2960/33,5 | 51/3500/36 | 48 /3300/33 | --/--/-- | 48/2950/33 |
|  |  |  |  |  |  |  |  |  |  |
| **Milestones** |  |  |  |  |  |  |  |  |  |
| walking (m) | 18 | 12 | 10 | 14 | 14 | 13 | 13 | 18 | 24 |
| first words (m) |  | 18 | 18 | 14 |  | 24 | 13 |  | 48 |
| first sentence (m) | 48 | 30 | no functional language | 40 | no functional language | 54 | 26 | 36 | no language |
| **Main somatic comorbidities** | Alopecia areata Hair loss | no | Febrile seizures at day 2 post birth | no | no | no | no |  |  |
| **Main psychiatric comorbidities** |  | ADHD | no | Single phobia | ADHD | ADHD | Sleep-onset insomnia, ADHD | Panic disorder with agoraphobia, cannabis dependence |  |
| **CLINICAL EXAMINATION** |  |  |  |  |  |  |  |  |  |
| **Developmental Coordination Disorder *** | yes | yes | yes | yes | yes | yes | yes | NA | yes |
|  |  |  |  |  |  |  |  |  |  |
| **Ear Nose Throat examination** |  |  |  |  |  |  |  |  |  |
| Subjective features | painful hyperacusis | painful  hyperacusis | painful hyperacusis | painful  hyperacusis | painful  hyperacusis | painful hyperacusis | painful hyperacusis | auditory hallucinations | painful  hyperacusis |
| Auditory acuity tests | normal range |  | normal range | normal range | normal range | normal range | normal range | normal range | non compliant |
| Auditory brainstem response test | normal range |  | shortened wave I-V interpeak latency | shortened wave I-V interpeak  latency | shortened wave I-V interpeak  latency | normal range | shortened wave I-V interpeak latency | wave I-V interpeak latency shortened | non compliant |
| **FAMILY HISTORY** |  |  |  |  |  |  |  |  |  |
| Father |  | sluttering |  | ASD without ID |  |  | chronic motor tic disorder | GAD |  |
| Mother |  | dyslexia/  dysgraphia |  |  | MDD, anxiety disorder |  | MDD | GAD |  |
| Siblings, other members | One daughter with ID carrying the deletion | Cousin with epilepsy |  | One brother with ASD but without ID not carrying the deletion |  |  | One brother with ASD and ID not carrying the variant | Two brothers, both with ASD without ID all carrying the variant |  |

GAD: generalized anxiety disorder; IQ: intellectual quotient; MDD: mood disorder; RPM: Raven’s Progressive matrices, m: male; f: female; nv: non verbal;

* based on the Neurological exam, the ADI-R and the Developmental Coordination Disorder Questionnaire

**Supplementary Table S9. Auditory brainstem responses in patients and their relatives.**

|  | **LEFT EAR** | | | | | **RIGHT EAR** | | | | |
| --- | --- | --- | --- | --- | --- | --- | --- | --- | --- | --- |
|  | Frequency: 29 clicks / sec | | | | | Frequency: 29 clicks / sec | | | | |
|  | Affected Carriers  (mean/SD/N) | Unaffected carriers  (mean/SD/N) | Unaffected non carriers  (mean/SD/N) | KW(2df) χ2 | p | Affected Carriers  (mean/SD/N) | Unaffected carriers  (mean/SD/N) | Unaffected non carriers  (mean/SD/N) | KW(2df) χ2 | p |
| Intensity: 80 dB |  |  |  |  |  |  |  |  |  |  |
| latency V | 5.50/0.38/6 | 5.55/0.45/7 | 5.41/0.21/5 | 0.51 | 0.78 | 5.47/0.39/7 | 5.75/0.46/7 | 5.44/0.38/8 | 1.89 | 0.39 |
| latency III-V | 1.88/0.22/5 | 1.90/0.24/6 | 1.78/0.18/5 | 1.56 | 0.46 | 2.03/0.36/5 | 2.02/0.63/7 | 1.65/0.15/7 | 4.26 | 0.12 |
| latency I-V | 3.96/0.43/4 | 4.14/0.33/6 | 3.86/0.14/5 | 2.22 | 0.33 | 4.23/0.21/5 | 4.11/0.48/7 | 3.72/0.49/7 | 4.63 | 0.09 |
| latency I-III | 2.06/0.22/4 | 2.23/0.19/6 | 2.07/0.15/5 | 2.17 | 0.34 | 2.30/0.21/5 | 2.09/0.40/7 | 2.17/0.10/6 | 2.19 | 0.33 |
| latency III | 3.41/0.52/5 | 3.78/0.2/6 | 3.75/0.19/5 | 2.55 | 0.28 | 3.48/0.46/4 | 3.62/0.37/7 | 3.65/0.28/6 | 0.33 | 0.84 |
| latency I | 1.25/0.33/4 | 1.54/0.12/6 | 1.65/0.32/5 | 4.74 | 0.09 | 1.30/0.30/5 | 1.53/0.13/7 | 1.61/0.15/7 | 4.21 | 0.12 |
| Intensity: 60 dB |  |  |  |  |  |  |  |  |  |  |
| latency V | 5.94/0.61/9 | 6.61/0.74/5 | 6.12/0.44/8 | 4.18 | 0.12 | 5.89/0.32/9 | 6.33/0.29/5 | 5.95/0.45/8 | 4.72 | 0.09 |
| latency III-V | 1.90/0.30/7 | 2.05/0.07/2 | 1.79/0.27/5 | 2.67 | 0.26 | 2.00/0.25/7 | 1.93/0.21/4 | 1.71/0.16/6 | 5.18 | 0.08 |
| latency I-V | 3.88/0.24/6 | 4.60/0.32/2 | 4.20/0.32/6 | 5.87 | 0.05 | 3.94/0.48/4 | 3.10/0.25/3 | 3.77/0.32/8 | 1.63 | 0.44 |
| latency I-III | 2.13/0.18/6 | 2.27/0.00/1 | 2.16/0.33/5 | 0.21 | 0.90 | 2.10/0.41/4 | 2.21/2.3/3 | 0.81/2.90/6 | 2.51 | 0.29 |
| latency III | 4.03/0.70/7 | 3.95/0.11/2 | 4.04/0.52/4 | 0.17 | 0.92 | 3.91/0.14/7 | 4.55/0.43/4 | 4.10/0.45/6 | 4.76 | 0.09 |
| latency I | 1.80/0.14/6 | 1.72/0.07/2 | 1.83/0.32/6 | 0.99 | 0.61 | 1.90/0.18/4 | 2.52/0.20/3 | 2.04/0.35/8 | 5.62 | 0.06 |

**Supplementary Figure S1. Genetic and clinical characterization of the family AUDIJ001 and AUDIJ002 carrying CNTN5 CNVs.** **Family AUDIJ001 (a)** The CNTN5 deletion was transmitted from the proband with ASD to her daughter with specific language impairment (SLI). **(b)** Agilent array-CGH 180K profile of the daughter showing the 11q21q22.1 deletion (4.1 Mb) encompassing all the exons of CNTN5 (chr11: 96,144,810-100,268,562). JRKL antisense RNA 1 is deleted, but not MAML2. **(c)** Inverse DAPI FISH image: the control probe RP11-176D23 (green) spanning CNTN5 confirmed the heterozygous 11q21q22.1 deletion. Arrows indicate both chromosomes 11. **Family AUDIJ002 (d)** The proband with ASD and ADHD carried 5 copies of CNTN5 inherited from her mother diagnosed with specific learning disorder (SLD, reading). **(e)** Interphase FISH (Fluorescent In Situ Hybridization) image of the 5 copies of CNTN5 in the proband (probe RP11-418D05 in green) on chromosome 11 (the probe RP11-583A17 in 11p15.5 is labeled in red). **(f)** Agilent array-CGH (Comparative Genomic Hybridization) 180K profile of the daughter showing the 5 copies of CNTN5 (chr11:96,145,010-100,305,324).

**Supplementary Figure S2. CNTN5 and CNTN6 copy-number variants identified in patients with ASD.** CNTN5 and CNTN6 CNVs were detected using the HumanOmni1 Illumina array. For each SNP, a green dot represents the B Allele Frequency (BAF) and a red dot, the log R ratio (LRR) of hybridization intensity. The blue line shows the predicted number of copies (CN). Black-filled symbols indicate a diagnosis of ASD with intellectual disability, while grey-filled symbols indicate a diagnosis of ASD without intellectual disability. In multiplex families, an arrow indicates the proband. Based on the ADI-R and clinical examination, normal (green) or abnormal (red) hypersensitivity to sounds and motor coordination ability are indicated by a schematic ear and hand, respectively. The absence of ear/hand means that information was not available. All exonic CNVs of CNTN5 or CNTN6 identified in patients and controls are shown in Supplementary Figure S3.

**Supplementary Figure S3. Localization of the exonic CNVs (> 30kb) affecting CNTN5 (a) or CNTN6 (b) in patients and controls.** HBAC: Health Aging and Body Composition cohort; KORA: KORA ("Kooperative Gesundheitsforschung in der Region Augsburg" which translates as "Cooperative Health Research in the Region of Augsburg"); COGEND: Collaborative Genetic Study of Nicotine Dependence

**Supplementary Figure S4. Families with CNTN5 and CNTN6 coding SNVs from the PARIS cohort.** The patients with ASD from the PARIS cohort (N=212) were investigated using Sanger sequencing. Coding-sequence variants are represented by dots (two dots = homozygous state). Black-filled symbols indicate a diagnosis of ASD with intellectual disability, while grey-filled symbols indicate a diagnosis of ASD without intellectual disability. In multiplex families, an arrow indicates the proband. Based on the ADI-R and clinical examination, normal (green) or abnormal (red) hypersensitivity to sounds and motor coordination ability are indicated by a schematic ear and hand, respectively. The absence of ear/hand means that information was not available. The scores for Social Responsiveness Scale are indicated when available. A total score of 76 or higher (red) is considered severe and strongly associated with clinical diagnosis of Autistic Disorder. Scores of 60 through 75 (Orange) are interpreted as indicating mild to moderate deficiencies in reciprocal social behavior. Scores of 59 and below (green) are considered to be within typical limits and generally not associated with clinically significant ASD. A question mark means that DNA was unavailable. The family AU-SWE-162 carried the CNTN5^L790I^ and the CNTN6^R303Q^.

**Supplementary Figure S5. Families with CNTN5 and CNTN6 coding SNVs from Canada.** The cohort of patients with ASD from Canada (N=289 families) was investigated using whole genome sequencing. Coding-sequence variants are represented by dots. Black-filled symbols indicate a diagnosis of ASD with intellectual disability, while grey-filled symbols indicate a diagnosis of ASD without intellectual disability. For some individuals there was no information on IQ. Based on the ADI-R and clinical examination, normal (green) or abnormal (red) hypersensitivity to sounds is indicated by a schematic ear. The absence of ear means that information was not available.

**Supplementary Figure S6. Frequency of CNTN6 private SNVs in this study, in Murdoch et al. PLOS Genetics (2015) and in the ExAC cohort.** The SNVs with a single allele count in the ExAC cohort can be found at [*http://exac.broadinstitute.org/*](http://exac.broadinstitute.org/). Private mutations in this study and in Murdoch et al. (2015) met the following criteria: seen only once in either cases or controls exclusively, missense, nonsense, splice site, or start or stop codon disruptions with a frequency of less than 1% in the ExAC database.

**Supplementary Figure S7. Venn diagram of the ASD-risk genes from different databases.** The ASD genes were taken from the Class I-III genes of Yuen et al. (2015) [^1^](#_ENREF_1), the TADA genes from Sanders et al. (2015) [^2^](#_ENREF_2) or from the SFARI database (https://gene.sfari.org/autdb/Welcome.do). The complete list of genes is available as an excel file in the supplementary Table S10. The 37 genes present in the 3 databases are: NRXN1, SHANK3, KDM5B, WDFY3, KDM6B, NCKAP1, ASH1L, PTEN, TCF7L2, DYRK1A, BCL11A, GRIN2B, SYNGAP1, ADNP, ANK2, ETFB, APH1A, CTTNBP2, CUL3, NAA15, KATNAL2, NLGN3, MIB1, SUV420H1, TBR1, SHANK2, MBD5, CHD8, ARID1B, POGZ, SETD5, TRIO, KMT2C, MYT1L, GABRB3, SCN2A, FOXP1.

**Supplementary Figure S8. Impact of the CNTN6 variants on neurite outgrowth.** Cultures of cortical neurons were prepared using newborn rats (P0-P1) as described in Materials and Methods. **a.** The length of the longest neurite (mean +/- SEM) was measured in two independent experiments (1 and 1’). Neurons were co-cultured with HEK cells expressing the empty vector (EV), the wild-type CNTN6 (WT) or the CNTN6 carrying the variants identified in patients only (red), in patients and controls (orange) or in controls only (green). The intervals for the length of the longest neurite for experiments performed with the EV or the CNTN6 WT are indicated by the light and dark grey bars, respectively. The numbers of neurons analyzed are indicated in the graph. Statistical analyses were performed by using a F test to compare variances, followed by an impaired t-test with Welch correction. **b.** Representative western blots of selected co-culture supernatants for the detection of CNTN6 protein. No CNTN was detected with the empty vector (EV). Western blot of CNTN6 WT, CNTN6^S57L^ show a major band at the expected molecular weight of 130 KDa. For CNTN6^T958I^, we observed a major band at 260KDa suggesting a dimeric form of the protein.

**Supplementary Figure S9. Effect of CNTN6 variants on protein structure. (a)** Structure of the immunoglobulin domains. Models of wild-type and mutated human CNTN6 Ig1-4 are superimposed, the variants are mapped in spheres. Close views highlight the impact of CNTN6^G310S^ on the structure. (**b)** Structure of the fibronectin domains. Each Protein Data Bank (PDB) ID used for the 3D-BLAST is indicated.

**References**

1. Yuen RK, Thiruvahindrapuram B, Merico D, Walker S, Tammimies K, Hoang N et al. Whole-genome sequencing of quartet families with autism spectrum disorder. Nat Med 2015; 21: 185-191.FORMAT

2. Sanders SJ, He X, Willsey AJ, Ercan-Sencicek AG, Samocha KE, Cicek AE et al. Insights into Autism Spectrum Disorder Genomic Architecture and Biology from 71 Risk Loci. Neuron 2015; 87: 1215-1233.FORMAT
