# Supplementary material for: CNTN6 mutations are risk factors for abnormal auditory sensory perception in autism spectrum disorders
Source: Mol Psychiatry. 2016 May 10;22(4):625–33. doi: 10.1038/mp.2016.61 (PMC5378808; doi:10.1038/mp.2016.61)
Supplement: Supplementary Table Legends [file mp201661x2.doc]

**Supplementary Table S10. ASD-risk genes from different databases.** The ASD genes were taken from the Class I-III genes of Yuen et al. (2015), the TADA genes from Sanders et al. (2015) or from the SFARI database (https://gene.sfari.org/autdb/Welcome.do).

**Supplementary Table S11. CNVs affecting exons of *CNTN5* or *CNTN6* genes in the BBGRE database.**

All data were taken from the Brain & Body Genetic Resource Exchange (BBGRE version 3.0; https://bbgre.brc.iop.kcl.ac.uk/) database

**Supplementary Table S12. CNVs affecting exons of *CNTN5* or *CNTN6* genes in the DECIPHER database.** All data were taken from the Decipher database (https://decipher.sanger.ac.uk/index)
